# Antiparasitic activity of the steroid-rich extract of *Schima wallichii* against poultry cestode

**DOI:** 10.14202/vetworld.2024.1299-1306

**Published:** 2024-06-19

**Authors:** Pawi Bawitlung Lalthanpuii, Kholhring Lalchhandama

**Affiliations:** DBT-BUILDER National Laboratory, Department of Life Sciences, Pachhunga University College, Aizawl, Mizoram, India

**Keywords:** anthelmintic, cestode, medicinal plant, parasite, scanning electron microscopy

## Abstract

**Background and Aim:**

*Schima wallichii* Korth., commonly known as the needlewood tree (family Theaceae) has therapeutic uses in traditional Mizo medicine for human helminthiasis and serves as a balm against ectoparasites in animals. Although the medicinal properties have been studied experimentally, its use as a traditional anthelmintic remains unexplored. This study aimed to analyze the chemical components and antiparasitic activity of *S. wallichii*.

**Materials and Methods:**

The chemical analysis of *S. wallichi* bark extracts was conducted focusing on the secondary metabolites using petroleum ether, chloroform, and methanol. Gas chromatography-mass spectrometry (GC-MS) was used to identify the specific compounds. An anthelmintic susceptibility test was carried out against *Raillietina tetragona*, intestinal cestode parasite of fowl.

**Results:**

The methanol extract yielded the highest concentrations of alkaloids, carbohydrates, glycosides, sterols, saponins, and tannins among all the extracts. Sterols were the most abundant compounds in all extracts, with flavonoids being absent. Secondary metabolites were largely absent in the petroleum ether and chloroform extracts. The GC-MS data identified cholest-22-ene-21-ol as the major steroid component. The cestode parasite was inhibited in a concentration-dependent manner by the plant extract. The plant extract’s anthelmintic activity was evident through observable damage to the parasite’s outer structure.

**Conclusion:**

Phytosterols in *S. wallichii* bark are responsible for its anthelmintic properties. The mechanism and pharmaceutical properties of the anthelmintic molecule require further exploration.

## Introduction

The global health crisis resulting from anthelmintic drug resistance in animals has been largely influenced by the advancements and discoveries of synthetic pharmaceuticals [1]. Managing veterinary helminths is a complex issue due to their intricate life cycles, which involve multiple hosts and unidentified molecular interactions [2]. Due to parasite resistance, major anthelmintics cause significant losses in the animal industry [3]. Anthelmintics face resistance due to helminths’ sophisticated mechanisms, such as enhanced elimination, accelerated metabolism, reduced receptor binding, decreased receptor expression, and diminished drug affinity [4]. The development of new, broadly applicable drugs has been hindered [5].

One possible source for new anthelmintic compounds lies in the use of plants and their derivatives in traditional medicines [6, 7]. Although comprehensive reports have chronicled traditional systems’ screening of plants for anthelmintic activity [8, 9], pharmaceutical ventures have largely neglected research in medicinal plants [1]. The precise medicinal sources, lack of experimental validations, and difficulty in isolating bioactive compounds are major challenges in medicinal plant research [10]. It is imperative that traditional uses of individual plants be subjected to systematic investigations and analyses [11].

*Schima wallichii* Korth., commonly known as the needlewood tree, is a tree species belonging to the Theaceae family. It originates in Asian countries such as China, India, Indonesia, Nepal, and Taiwan [12, 13]. This medicinal plant, widely known for its antibacterial, antifungal [14], anti-inflammatory, analgesic [15, 16], and cellular protective properties [13], is recognized for the healing benefits of its bark extract. The anticancer and antimalarial properties of the plant can be attributed to the presence of kaempferol-3-O-rhamnoside, a flavone glycoside isolated from its leaves. This compound inhibits breast cancer cells [17] and *Plasmodium falciparum* [18]. The bark is used in India for fever, bacterial infections, and wound treatment [19]. Mizoram in Northeast India, the most distant state inhabited by the Mizo people, lies within the Indo-Burma biodiversity hotspot [20]. In Mizo traditional medicine, the extracts from the bark and leaves of *S. wallichii* are used to expel blood parasites, intestinal worms, and external parasites [12].

Based on its anthelmintic properties, this study aimed to investigate the effect of the plant extract on intestinal parasitic cestodes and identify the active compounds.

## Materials and Methods

### Ethical approval

The study used cestode parasites collected from local fowl and was approved by the Institutional Animal Ethics Committee of Pachhunga University College (PUC-IAEC-2016-Z2).

### Study period and location

The study was conducted from April 2020 to October 2023 at Pachhunga University College, Aizawl, India.

### Plant specimen and extraction

The plant specimen, *S. wallichii* parts were collected from Pachhunga University College campus in Aizawl, Mizoram, India, located at 23.7233° N, 92.7271° E. The fresh leaves and flowers of *S. wallichii* were prepared for herbaria and authenticated at the Botanical Survey of India, Eastern Regional Office, Shillong, Meghalaya. Voucher specimens were deposited under the accession code PUC-S-18-01 in the herbarium collection of Pachhunga University College. The barks were peeled off, cleansed in water, chopped into fine pieces, and kept in shade to dry for 4 weeks. Batches of 540 g of dried samples were extracted in a Soxhlet apparatus using solvents of different polarities, such as petroleum ether (having a polarity index of 5.1), chloroform (polarity index of 4.1), and methanol (polarity index of 0.1). The entire extraction was run for 72 h in each solvent. The crude extracts were concentrated by recycling the solvent in a vacuum rotatory evaporator, Buchi Rotavapor® R-100 (Flawil, Switzerland). The final extracts were refrigerated at 4°C until their use in chemical and biological assays.

### Phytochemical tests

The compounds present in *S. wallichii* bark extracts were identified using eight standard phytochemical tests [21]. Different tests, including Dragendorff’s, Hager’s, Mayer’s, and Wagner’s for alkaloids; Barfoed’s, Benedict’s, Fehling’s, and Molisch’s for carbohydrates; Shinoda’s and zinc hydrochloride (ZnCl_2_) for flavonoids; Baljet and Legal’s for glycosides; Liebermann-Burchard’s and Salkowski for phytosterols; Benedict’s and Fehling’s for reducing sugars; foam for saponins; ferric chloride (FeCl_3_), potassium dichromate (K_2_Cr_3_O_7_), and lead acetate (Pb(C_2_H_3_O_2_)_2_) for tannins, were used to identify various compounds.

### Gas chromatography-mass spectrometry (GC-MS)

The Perkin Elmer AutoSystem™ XL chromatograph with TurboMass™ spectrometer (Waltham, USA) was used for analyzing the chemical composition of the methanol extract of *S. wallichii* bark. The plant extract was injected into an Elite-5MS capillary column (30 m length × 0.25 mm inside diameter × 0.25 μm thick) after being dissolved in acetonitrile. The injector temperature was maintained at 260°C and the oven temperature rose from 75°C to 280°C, increasing by 10°C each minute. 2 μL of the sample, split in 1:50, was introduced into the gas stream at a flow rate of 1 mL/min using helium as the carrier gas. The mass spectrometer was operated at a temperature of 220°C. From the National Institute of Standards and Technology’s database, compounds were identified using their retention times, chemical compositions, and molecular weights.

### Anthelmintic susceptibility assay

Anthelmintic susceptibility was assessed on a cestode, *Raillietina tetragona* Molin, 1858, following the method of helminth survival assay [22]. From the intestines of the locally-sourced chickens, *Gallus gallus* Linnaeus, 1758, the cestode parasites were obtained. They were treated with plant extracts and albendazole at 20 mg/mL in a 37 ± 1°C incubator. 0.9% phosphate-buffered saline (PBS) with 1% differential concentration of dimethyl sulfoxide (DMSO) served as the negative control. The antiparasitic effectiveness was evaluated based on survival in culture media. Student’s *t*-test was used with a significance level of p < 0.05 to analyze the data.

### Scanning electron microscopy (SEM)

SEM analysis was performed on *R. tetragona* exposed to the plant extract using established protocols for helminth parasites [23]. The cestodes were preserved in 10% neutral-buffered formaldehyde at 4°C for 4 h. The dehydration process was carried out with increasing concentrations of acetone. The samples were dried in a chamber at 25°C after being treated with tetramethylsilane. The JSM-6360 SEM (JEOL Ltd., Tokyo, Japan), operated at an electron accelerating voltage of 20 kV, was used to examine the samples after they were coated with gold using the JFC-1100 instrument (JEOL Ltd.).

## Results

### Extraction and detection of phytocompounds

The bark extracts of *S. wallichii* yielded progressively higher percentages with increasing solvent polarity (Table-1): 0.29% petroleum ether, 0.48% chloroform, and 32.17% methanol. The polarity of phytocompounds influenced the number of compound groups present, as indicated by qualitative tests (Table-2). The petroleum ether extract contained carbohydrates, phytosterols, and tannins but was deficient in most secondary metabolites. The chloroform extract held carbohydrates, phytosterols, reducing sugars, and saponins. The methanol extract excluded flavonoids in the tested compound groups. Phytosterols were the most abundant compounds in all extracts, while tannins were also universally detected.

**Table-1:** Yield of extraction of *Schima wallichi* bark using different solvents.

Solvent	Polarity index	Weight of sample (g)	Extractive weight (g)	Extractive value (%)
Petroleum ether	0.1	540	1.55	0.29
Chloroform	4.1	540	2.58	0.48
Methanol	5.1	540	173.69	32.17

*S. wallichi=Schima wallichi*

**Table-2:** Phytochemical groups detected in *S. wallichi* bark extracts.

S. No.	Phytochemicals	Name of test	Petroleum ether extract	Chloroform extract	Methanol extract
1.	Alkaloids	Dragendorff’s test	-	-	+
		Hager’s test	-	-	+
		Mayer’s test	-	-	+
		Wagner’s test	-	-	+
2.	Carbohydrates	Barfoed’s test	-	-	+
		Benedict’s test	-	+	+
		Fehling’s test	-	+	+
		Molisch’s test	+	+	+
3.	Flavonoids	Shinoda’s test	-	-	-
		ZnCl_2_ test	-	-	-
4.	Glycosides	Baljet test	-	-	+
		Legal’s test	-	-	-
5.	Phytosterols	Liebermann-Burchard’s test	+	+	+
		Salkowski test	+	+	+
6.	Reducing sugars	Benedict’s test	-	-	+
		Fehling’s test	-	+	+
7.	Saponins	Foam test	-	+	+
8.	Tannins	FeCl_3_ test	-	+	+
		K_2_Cr_3_O_7_ test	-	-	+
		Pb (C_2_H_3_O_2_)_2_ test	+	+	+

-=Indicates absence, +=Indicates presence, *S. wallichi*=*Schima wallichi*, ZnCl_2_=Zinc hydrochloride, FeCl_3_=Ferric chloride, K_2_Cr_3_O_7_=Potassium dichromate, Pb (C_2_H_3_O_2_)_2_=Lead acetate

### Chemical components

**Figure-1:**
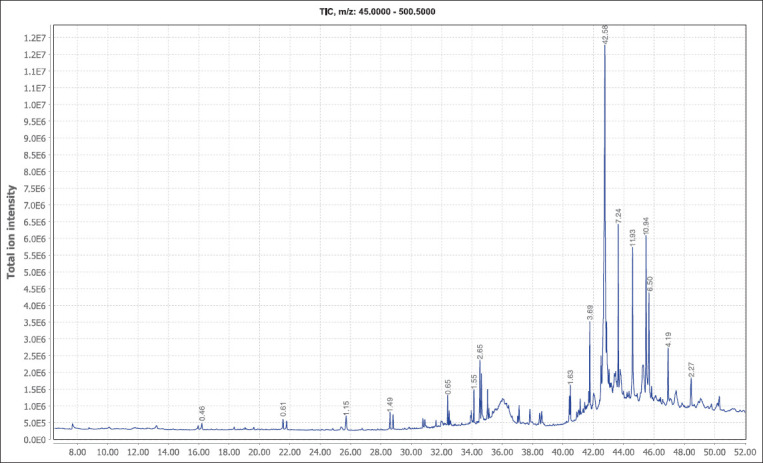
Gas chromatogram of the methanol extract of *Schima wallichii* bark.

GC-MS analysis was conducted on *S. wallichii* bark’s methanol extract, which contained the most phytocompounds and exhibited the strongest activity against cestode parasites. The chromatogram and mass spectra indicate that the plant extract is primarily composed of lipids, alkanes, and terpenes (Table-3). Sixteen major peaks in the chromatogram were accounted for by nine different compounds (Figure-1). About 42.58% of the total detected compounds were identified as the steroid cholest-22-ene-21-ol (3,5-dehydro-6-methoxy-pivalate-cholest-22-ene-21-ol). At five different retention times, dotriacontane, the second-most abundant compound, was identified as an alkane.

**Table-3:** GC-MS profiling of the methanol extract of *S. wallichi*i bark.

Peak	Retention time (min)	Abundance %	Compound	Formula	Molecular weight
1	16.203	0.46	Dodecane	C_12_H_26_	170.33
2	21.553	0.61	1-Pentadecene	C_15_H_30_	210.39
3	25.709	1.15	2,4-Di-tert-butylphenol	C_14_H_22O_	206.32
4	28.600	1.49	1-Pentadecene	C_15_H_30_	210.39
5	32.500	0.65	Heneicosane	C_21_H_44_	296.57
6	34.129	1.55	Palmitic acid	C_17_H_34_O_2_	270.45
7	34.627	2.65	Palmitic acid	C_17_H_34_O_2_	270.45
8	40.488	1.63	Dotriacontane	C_32_H_66_	450.87
9	41.766	3.69	Dotriacontane	C_32_H_66_	450.87
10	42.760	42.58	Cholest-22-ene-21-ol	C_35_H_54_O_3_	498.48
11	43.641	7.24	β-Amyrin	C_30_H_50_O	426.72
12	44.588	11.93	Dotriacontane	C_32_H_66_	450.87
13	45.471	10.94	(Z)-13-docosenamide	C_22_H_43_NO	337.58
14	45.665	6.50	Dotriacontane	C_32_H_66_	450.87
15	46.927	4.19	Dotriacontane	C_32_H_66_	450.87
16	48.441	2.75	Tetrapentacontane	C_54_H_110_	759.45

*S. wallichi*=Schima wallichi, GC-MS=Gas chromatography-mass spectrometry

### Anthelmintic activity

The *R. tetragona* cestodes thrived in PBS with 1% DMSO in a 37°C incubator for 3 days, no food was added. The cestodes’ susceptibility to albendazole and the methanol extract of *S. wallichii* is demonstrated in Table-4. 20 mg/mL albendazole served as the positive anthelmintic reference. The *S. wallichii* bark extract inhibited the test cestode in a concentration-dependent manner, as suggested by the relative (control group survival-normalized) survival values. The plant extract and albendazole were similar in their inhibitory effect at 20 mg/mL (albendazole: 3.85 ± 0.69 h, plant extract: 4.57 ± 1.15 h).

**Table-4:** Anthelmintic efficacy of *S. wallichi*i bark extract against the cestode, *R. tetragona*.

Treatment media	Dose (mg/mL)	Normalized survival time (h) in mean±SD	*t*-value	*t*-critical value
Negative control	0	100.00±2.57	NA	NA
Albendazole	20	003.85±0.69*	88.39	2.45
*S. wallichi*i	20	004.57±1.15*	82.90	2.36

* Significantly different at *P <* 0.05 against negative control at *n* = 6; NA=Not applicable, SD=Standard deviation, *S. wallichi*=*Schima wallichi*, *R. tetragona=Raillietina tetragona*

### SEM

**Figure-2:**
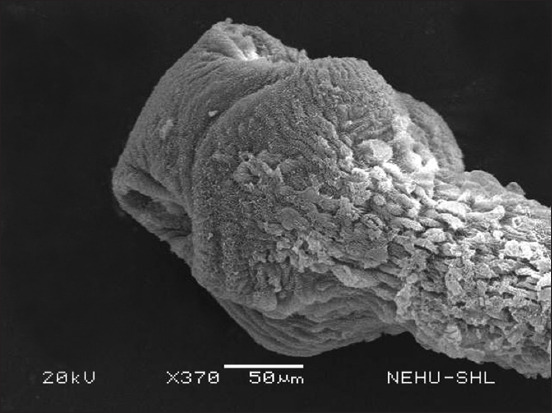
Scanning electron micrograph of *Raillietina tetragona* treated with *Schima wallichii* bark extract. The portion shows the anterior region, the scolex. Ear-like structures on either side are suckers, the attachment organs. Warty projections are eruptions of the tegument.

**Figure-3:**
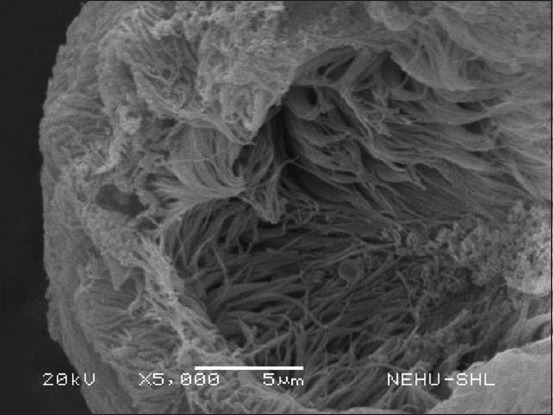
A magnified view of the scolex showing a single sucker. Numerous thread-like filaments are fragmented spines.

After *S. wallichii* bark extract treatment, *R. tetragona* exhibited significant structural alterations as observed through SEM. The effects of the plant extract at a concentration equivalent to 20 mg/mL albendazole were selected. The entire body displayed signs of damage. In Figure-2, the base of the scolex is expanded, while the sucker regions are constricted. The tegument, or general body surface, appeared fuzzy, suggesting the loss of microtriches, its hair-like projections. On closer examination of a sucker (Figure-3), the piercing spines of the parasite’s attachment organs appeared fragmented and fragile.

**Figure-4:**
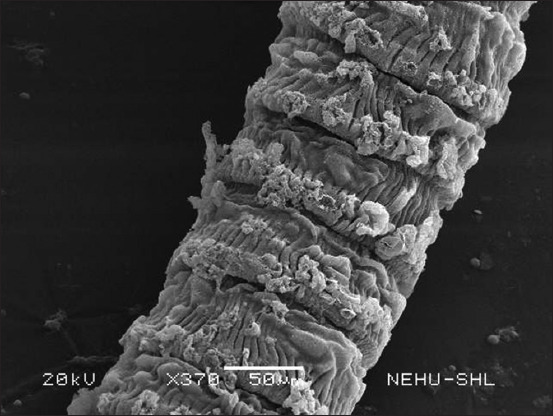
The neck region of *Raillietina tetragona* treated with *Schima wallichii* bark extract showing the immature body segments, proglottids, near the scolex. Conspicuous warts and eruptions are visible on every segment.

**Figure-5:**
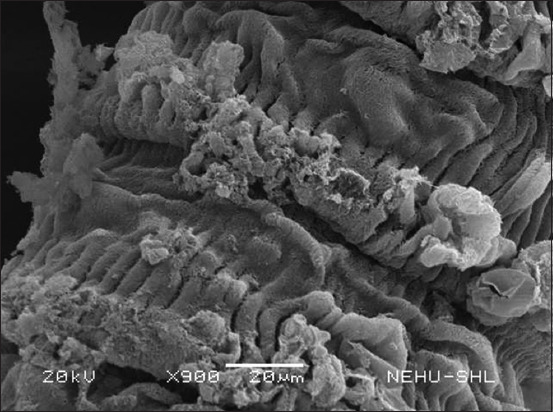
A magnified view of the immature proglottids. Tegumental eruptions are associated with erosion of the microtriches.

**Figure-6:**
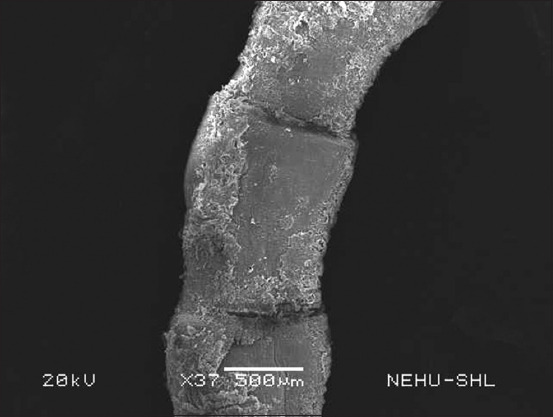
The posterior region of *Raillietina tetragona* treated with *Schima wallichii* bark extract showing the egg-containing gravid proglottids. Much of the tegumental surface is eroded.

**Figure-7:**
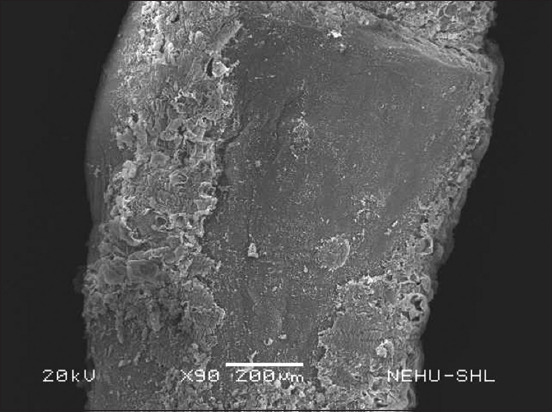
A magnified view of a single gravid proglottid. Tegumental erosion is seen on most of the surface area, the remaining microtriches are clumped and form flakes.

The cestode’s main body consists of a chain of proglottids. Several warts and protrusions were evident in the anterior neck region of the proglottids (Figure-4). Figure-5 shows abnormalities appearing as eruptions, blebs, and erosions. The microtriches are showing signs of deterioration. The extensively damaged gravid proglottids had visible similarities to the warts and eruptions found on the posterior egg-containing segments (Figure-6). The microtriches on the tegument were completely eroded (Figure-7). The microtriches formed disheveled clumps of tegumental flakes.

## Discussion

Important phytocompounds are present in the bark of *S. wallichii*. The plant-derived steroid, cholest-22-ene-21-ol, is known to exist in select plant species. The active compound found predominantly in *Ehretia serrata* leaves is responsible for its anti-asthmatic, anti-epileptic, anti-malarial, antimicrobial, and wound-healing effects [24]. The *Halomonas* bacteria contain the sterol as an antibacterial component that acts through induction of biofilm formation [25]. The anti-toxic effects of *Ajuga parviflora* leaf extract in the liver of experimental rats are attributed to the sterol [26]. The antibacterial activities of *Sargassum crassifolium*, which contains it, have been experimentally proven [27]. β-amyrin, another ubiquitous triterpenoid identified in this study, possesses antibacterial [28], antinociceptive [29], anti-inflammatory [30], antihyperglycemic, and hypolipidemic [31] properties.

*Raillietina* species are common and abundant helminth parasites of fowl and are, thereby, convenient laboratory models in anthelmintic susceptibility tests because of their availability and ease of maintenance in culture media [23, 32]. These tapeworms have typical cestode features, including flat, segmented bodies. Cestodes have simpler morphological and anatomical structures than other helminths, with fewer external body parts, nervous tissues, and digestive organs. The tegument, their body surface, performs multiple functions, such as protection, nutrient absorption through passive diffusion, and sensory activities [33, 34]. Thousands of microtriches, uniformly covering the tegument, are responsible for these functions. Anthelmintics specifically target the tegument, microtriches, and underlying subtegumental tissue of parasitic worms, causing damage to these structures [35, 36].

In cestode infections, albendazole and other benzimidazoles are recommended as the preferred drugs. These drugs led to the collapse of the rostellum, degeneration of microtriches, and blistering of the tegument in the human cestode *Echinococcus granulosus* [37]. The application of albendazole and praziquantel together led to alterations in the suckers, loss of spines, and overall distortion of the tegument along with microtrich disintegration in *E. granulosus* and *Mesocestoides corti* [38, 39]. The tegument of *Raillietina* *echinobothrida* underwent extensive contraction and collapsed following a single albendazole treatment, resulting in the absorption of suckers and the loss of microtriches [40].

Nitazoxanide led to tegumental deformities and notable reduction in microtriches in both *Echinococcus multilocularis* and *E. granulosus* [41]. Lonidamine and 6-aminonicotinamide led to body swelling, tegument erosion, rostellum disintegration, and sucker disintegration in *E. granulosus* and *E. multilocularis* [42]. Praziquantel caused constriction of the tegument, shrinkage of the suckers, dislocation of spines, and erosion of microtriches in *R. echinobothrida* [43]. Different anthelmintics cause distinct structural damages to the tegument. *S. wallichii* bark extract affects *R. tetragona*’s entire body surface. The anthelmintic substance’s unique impact was indicated by the occurrence of tegumental eruption and erosion. GC-MS data indicate that cholest-22-ene-21-ol is the primary candidate for the anthelmintic activity of the plant. This study bolsters the need for more research on *S. wallichii*’s molecular impacts and medicinal applications.

## Conclusion

The bark of *S. wallichii* contains lipids, alkanes, and terpenes. Phytosterol content was detected and confirmed through phytochemical screening and GC-MS analysis. The steroid, cholest-22-ene-21-ol was the likely cause of the observed biological activities. The *R. tetragona* cestode parasite was inhibited concentration-dependently by the plant extract. SEM showed extensive damage to the cestode’s tegument after treatment with the plant extract, indicating anthelmintic activity. Observed effects included the disintegration of spines, degeneration of microtriches, and extensive eruption and erosion of the tegument. The study demands exploration into the primary compounds and their distinct pharmacological functions.

## Authors’ Contributions

KLC: Conceived and designed the study, interpreted the data, and prepared the draft. PBL: Collected the materials, performed the experiments, and generated the data. Both authors have read, reviewed, and approved the final manuscript.
